# Global Deletion of ALDH1A1 and ALDH1A2 Genes Does Not Affect Viability but Blocks Spermatogenesis

**DOI:** 10.3389/fendo.2022.871225

**Published:** 2022-04-28

**Authors:** Traci Topping, Michael D. Griswold

**Affiliations:** School of Molecular Biosciences, College of Veterinary Medicine, Washington State University, Pullman, WA, United States

**Keywords:** ALDH1A1, ALDH1A2, retinoic, spermatogenesis, viability

## Abstract

The transition of undifferentiated A spermatogonia to differentiated spermatogonia requires the action of retinoic acid (RA). The synthesis of retinoic acid from retinal in the seminiferous epithelium is a result of the action of aldehyde dehydrogenases termed ALDH1A1, ALDH1A2, and ALDH1A3. We used a mouse with a global deletion of the *Aldh1a1* gene that is phenotypically normal and the *CRE*-loxP approach to eliminate *Aldh1a2* genes globally and from Sertoli cells and germ cells. The results show that global elimination of *Aldh1a1* and *Aldh1a2* genes blocks spermatogenesis but does not appear to affect viability. The cell specific elimination of *Aldh1a2* gene showed that retinoic acid synthesis by Sertoli cells is required for the initial round of spermatogonial differentiation but that there is no requirement for retinoic acid synthesis by germ cells. In both the global gene deletion and the cell specific gene deletions the maintenance of *Aldh1a3* activity could not compensate.

## Introduction

The active form of vitamin A is retinoic acid that is synthesized in precise cellular locations by a two-step mechanism. First, retinol which is the circulating form of the vitamin is oxidized in a reversible reaction to retinal by retinol dehydrogenase (RDH10). Retinal is then oxidized to retinoic acid in a nonreversible reaction by one of 3 retinal dehydrogenases known as ALDH1A1, ALDH1A2, and ALDH1A3 (1).

ALDH1A2 and ALDH1A3 are required during fetal development. ALDH1A2^−/−^ mice die during embryonic development and ALDH1A3^−/−^ mice die shortly after birth (2, 3). However, ALDH1A1^−/−^ mice develop normally (4). In humans, ALDH1A1 mRNA is found in the liver, kidney, testis, brain, lung, red blood cells, and lens of the eye while ALDH1A2 mRNA is found in the testis, uterus, and skeletal muscle, and ALDH1A3 mRNA is localized in the prostate, trachea, intestine, and testis (5). Clearly all three ALDH1A enzymes contribute to RA synthesis during postnatal life. All together these studies underscore the tissue-specific central roles that ALDH1A enzymes play in animal physiology, and the vital significance of obtaining information concerning the expression and essential nature of the activity of these enzymes in human tissues.

In the mouse testis, retinoic acid is essential for the progression of undifferentiated spermatogonia A to become differentiating spermatogonia A1 and enter into spermatogenesis (6). In the absence of retinoic acid, undifferentiated spermatogonia never begin this progression (7). We have previously shown that deletion of the RDH10 gene in Sertoli cells alone will inhibit the progression of undifferentiated spermatogonia and also the deletion in both germ cells and Sertoli cells blocks this progression (8). Enzyme inhibitors have been used to eliminate the activity of all 3 aldehyde dehydrogenases and using the *CRE*lox P approach all 3 Aldh1a genes have been deleted only in germ cells, only in Sertoli cells and in both cell types (9, 10). Both of these approaches have shown that the retinal dehydrogenases are essential for spermatogenesis and both enzymes are present in germ cells and Sertoli cells. Aldh1a1 is most highly expressed in the Sertoli cells and Aldh1a2 and Aldh1a3 are expressed primarily in the germ cells but all 3 enzymes appear to be expressed at some level in both cell types (11).

It has been known that global deletion of the *Aldh1a1* gene in mice has little effect and does not significantly alter fertility (4). Recent studies have shown that the knockout of Aldh1a2 alone or the simultaneous knockout of Aldh1a1-3 in germ cells has little effect on successful spermatogenesis and fertility (9, 12). However, the simultaneous knockout of Aldh1a1-3 in Sertoli cells does not allow the undifferentiated A spermatogonia to progress to differentiating A1 spermatogonia (9). In this study we have broadened these previous observations by examining the effect on spermatogenesis of leaving the *Aldh1a3* gene intact. We started with a mouse mutant with a global deletion in *Aldh1a1*. From that genotype we used the *CRE*-loxP system to remove the *Aldh1a2* gene in germ cells and/or Sertoli cells and globally in all cells. In addition, and we have included sperm counts and fertility studies.

## Materials and Methods

### Animal Care, Breeding and Genotyping

All procedures involving mice were approved by the Washington State University Committee on the Use and Care of Animals. The mouse colonies were maintained in a temperature-controlled environment with access to food and water *ad libitum*. Mice were euthanized by CO_2_ asphyxiation followed by cervical dislocation. Four mouse lines were generated for this study, each expressing Cre recombinases to inactivate the *Aldh1a2* gene in Sertoli cells, germ cells, both Sertoli and germ cells or globally. The *Aldh1a1* ^-/-^, *Aldh1a2* ^fl/fl^, *ERT-*Cre line was created by breeding *Aldh1a2* ^fl/fl^, *ERT*-Cre (12) with *Aldh1a1* ^-/-^ (a gift from John Amory and Jisun Paik at the University of Washington with permission from Jackson labs, JAX stock #012247). The offspring who were heterozygous for all 3 alleles were bred and those animals in the next generation who were homozygous for the 3 alleles were experimental animals. In every case the excision of the gene, *Aldh1a2* ^Δ^, was confirmed by genotyping (12).

The *Aldh1a1* ^-/-^, *Aldh1a2* ^fl/fl^, *Amh*-Cre^+^ line was created by initially breeding *Aldh1a1* ^+/-^ with *Aldh1a2* ^fl/fl^, to create mice that were *Aldh1a1* ^+/-^, *Aldh1a2* ^fl/-^. These were bred with mice carrying the *Amh*-Cre transgene (a gift from Marie-Claude Hofman, UT MD Anderson Cancer Center) and those progeny who were *Aldh1a1* ^+/-^, *Aldh1a2* ^fl/-^ and carrying the transgene were then bred together to accumulate the experimental and control mice *Aldh1a1* ^-/-^, *Aldh1a2* ^fl/fl^, *Amh*-Cre^+^ and *Aldh1a1* ^-/-^, *Aldh1a2* ^fl/-^, *Amh*-Cre^+^, respectively.

The experimental and control mice for the *Aldh1a1* ^-/-^, *Aldh1a2* ^fl/fl^, *Stra8*-Cre+ and *Aldh1a1* ^-/-^, *Aldh1a2* ^fl/fl^, *Stra8*-Cre+, *Amh*-Cre^+^ lines were generated from the same breeding scheme. Males carrying the *Stra8*-Cre transgene (13) were bred with *Aldh1a1* ^+/-^ females. Male offspring who were *Aldh1a1* ^+/-^ and carried the *Stra8*-Cre transgene were paired with *Aldh1a1* ^-/-^ females. Males from this breeding who were *Aldh1a1* ^-/-^, *Stra8*-Cre^+^ were bred with females, generated above, who were *Aldh1a1* ^-/-^, *Aldh1a2* ^fl/fl^, *Amh*-Cre^+^. Male offspring from this pairing who were *Aldh1a1* ^-/-^, *Aldh1a2* ^fl/-^, *Stra8*-Cre+, *Amh*-Cre^+^ were paired with females, *Aldh1a1* ^-/-^, *Aldh1a2* ^fl/fl^, *Amh*-Cre^+^, to generate experimental and control mice for both lines.

To determine the genotypes of the mice, PCR reactions were performed on template generated from a tail clip from each mouse. The primer sets for *Amh-*Cre and *ALDH1A1* are as follows: *Amh-Cre* forward primer GCGGTCTGGCAGTAAAAACTATC and reverse primer GTGAAACAGCATTGCTGTCACTT; *ALDH1A1* forward primer CAACCCTGAGCAAATCCTCCAC, reverse primer for the knockout TGGATGTGGAATGTGTGCGAG and reverse primer for wild-type GACAGATTGAGAGCAGTGTTTACCC. All others have been reported elsewhere (12).

### Fertility and Sperm Counts

Males with confirmed KO in germ cells or Sertoli cells or both germ and Sertoli cells or ERT-Cre, tamoxifen treated males and controls were aged to 7 weeks and then were paired with a female of known fertility for 2 months to assess fertility. At the end of the 2 months the males were euthanized for study and the females left for 3 more weeks to continue to monitor for litters. The number of offspring and number of litters for each male was recorded. Following this timeline, each male in this study was euthanized at approximately 4 months. The body was weighed immediately after euthanasia. One testis was placed in Bouin’s fixative for immunohistochemistry and one was detunicated, snap frozen and weighed. Both cauda epididymides were placed in DMEM at room temperature and processed for counting sperm. The cauda epididymides were cut into approximately 1mm^3^ pieces and incubated at 37°C for 15 minutes. Three µl of the sperm suspension was applied to a Cell Vision disposable counting slide (CV 1020-4CV) and analyzed using a SCACASA system (Fertility Technology Resources, Inc) following the manufacturer’s instructions. When the sperm numbers were over 80 million, the sperm suspension was diluted 4 fold with DMEM before counting the sperm.

### Histology

Bouin’s fixed testes were embedded in paraffin, cut into 4 µm sections and either stained with hematoxylin and eosin or immunohistochemistry was performed using primary antibodies to Stra8 (14).

### Tamoxifen Preparation and Administration

Tamoxifen (Sigma T5648) was dissolved in 10% ethanol and 90% sesame oil at a concentration of 10 or 20 mg/ml, and the solution was wrapped in aluminum foil to protect from light. Mice were injected intraperitoneally with 40 mg/kg tamoxifen once per day from postnatal day 8 to 10 and/or with 80 mg/kg tamoxifen for 5 days starting at day 21 postpartum. Alternatively, at day of birth and postnatal day 1, mice were injected intraperitoneally with 100 mg/kg tamoxifen dissolved in sesame oil only at a concentration of 5 mg/ml. Tamoxifen was stored for a maximum of one week at 4°C and warmed to room temperature before injections. To confirm that the action of tamoxifen on the *ERT*-Cre resulted in excision of the ALDH1A2 gene, *Aldh1a2* ^Δ^ genotyping was performed on tail clips collected after euthanasia.

### Retinoic Acid Injections

Retinoic acid (Sigma R2625) was made fresh each day in DMSO. For the mice expressing the *Stra8*-cre and/or *AMH-*cre, 10 µl of 20 mg/ml was intraperitoneally injected once at day 21. For the males expressing the *ERT*-cre, RA at a concentration of 10 mg/ml was injected intraperitoneally at a dose of 12.5 µg/g body weight once at day 21. Males were euthanized after one round of spermatogenesis, 42 days later. As a control the same volume of DMSO was injected at day 21.

## Results

**Figure 1:**
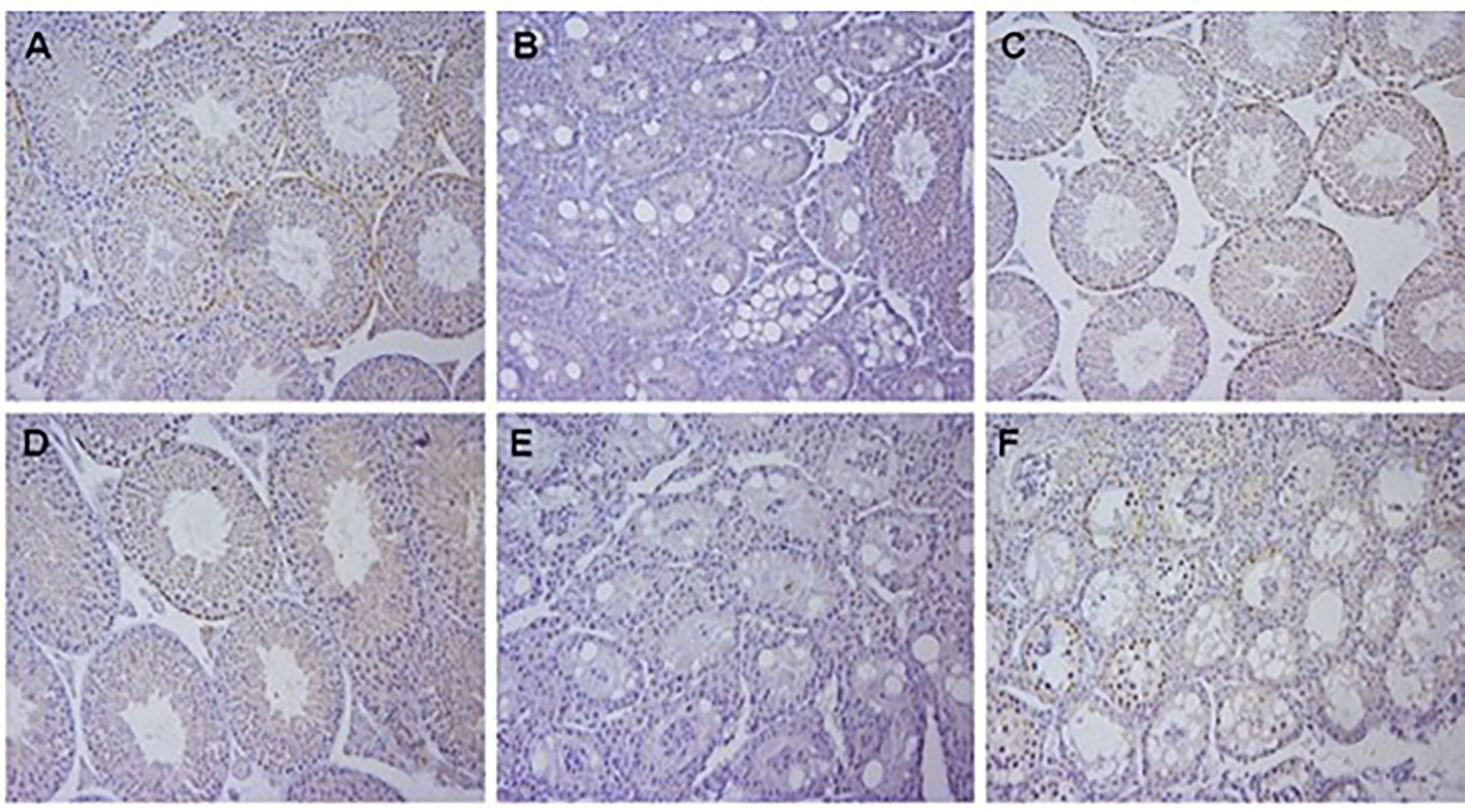
Histology of testis of mice with gene deletions in *Aldh1a1* and *Aldh1a2*. All samples were Bouin’s fixed, embedded in paraffin, cut into 4 µm sections and either stained with hematoxylin and eosin and immunohistochemistry was performed using primary antibodies to Stra8. **(A)** *Aldh1a1^-/-^, Aldh1a2^-/+^* 4 week old mice showing normal histology with sperm and Stra8 positive spermatogonia and preleptotene spermatocytes. **(B)** 4 week old mice with genotype of *Aldh1a1^-/-^, Aldh1a2^-/-^* after crossing mice in **(A)** with AMH-Cre mice resulting in deletion in Sertoli cells and a complete block at the progression of undifferentiated spermatogonia. **(C)** Mice with deletion in Sertoli cells as shown in **(B)** 42 days after a single injection of RA. Note recovery of normal spermatogenesis and partial synchrony of Stra8 expression. **(D)** 4 week old mice with genotype of *Aldh1a1^-/-^, Aldh1a2^-/-^* after crossing mice in **(A)** with Stra8-Cre mice resulting in deletion in germ cells. Note normal histology. **(E)** 4 week old mice with genotype of *Aldh1a1^-/-^, Aldh1a2^-/-^* after crossing mice in **(A)** with both AMH-Cre mice and Stra8-Cre mice resulting in deletion in both Sertoli cells and germ cells. **(F)** 4 week old mice with genotype of *Aldh1a1^-/-^, Aldh1a2^-/-^* after crossing mice with ERT-Cre mice and treatment with tamoxifen starting at postnatal day 8 and again at postnatal day 21 as described in the methods. Results illustrate the early block between A and A1 spermatogonia when RA is not available.

Using the *Aldh1a1^-/-^, Aldh1a2^fl/fl^* mice as our starting point we first wanted to see whether the presence of *Aldh1a3* altered the results from the previous studies of Teletin et al. (9). Their data showed that the deletion of all 3 Aldh1a genes in Sertoli cells blocked spermatogenesis at the conversion of A spermatogonia to A1 spermatogonia in mice. However, if these mice were injected with retinoic acid once, the block was removed, and spermatogenesis proceeded normally and continuously. They also showed that spermatogenesis was normal with the deletion of all 3 *Aldh1a* genes in germ cells alone. They concluded that RA from Sertoli cells was necessary for the initial A to A1 conversion of spermatogonia but that RA from germ cells could maintain the process. If RA synthesis was normal in Sertoli cells the presence of RA synthesis in germ cells was not necessary. We created *Aldh1a1^-/-^, Aldh1a2^fl/fl^* mice under control of *AMH Cre* or *Stra8 Cre* to produce deletions of only Aldh1a1 and Aldh1a2 in Sertoli cells or germ cells, respectively. *Aldh1a1^-/-^* mice have essentially normal spermatogenesis and we previously showed that *Aldh1a2^-/-^* mice also have normal spermatogenesis (12). However, the deletion of both of these two genes in Sertoli cells or germ cells recapitulated the results from deletion of all 3 genes reported by Teletin et al. (9). We also found that deletion of *Aldh1a1* and *Aldh1a2* genes in Sertoli cells completely blocked spermatogenesis at the conversion of A spermatogonia to A1 and that this block could be overcome by a single injection of RA (**Figure 1**). The knockout of genes coding for both enzymes in germ cells had no effect on sperm production. The results based on histology (**Figure 1**) were reflected in testis weights and number of sperm detected in the cauda epididymis. In breeding studies all of the crosses that had detectable sperm in the cauda produced normal litter numbers and sizes. So, the production of RA by Sertoli cells from either or both *Aldh1a1* or *Aldh1a2* enzymes is required for the initiation of spermatogenesis and the presence of *Aldh1a3* enzymes cannot compensate.

In order to examine the effect of globally deleting *Aldh1a1* and *Aldh1a2* we created *Aldh1a1^-/-^, Aldh1a2^fl/fl^* mice with an inducible ERT-Cre, and then utilized the injection of tamoxifen to activate the CRE activity. We used several protocols for the injection of tamoxifen. We injected once per day for 3 consecutive days starting at 8 days of age or once per day for 5 days at 21 days of age. In both protocols we found that spermatogenesis appeared to be little affected since the sperm counts per cauda epididymis were near normal and most of the mice fathered litters of near normal size. However, if we combined the protocols and injected tamoxifen for 3 consecutive days at 8 days of age and then repeated the injections for 5 days at 21 days of age, we found that by 4 to 5 months of age when the mice were analyzed the sperm counts went to zero and no litters were produced in breeding trials. Apparently, neither of the 2 individual tamoxifen injection regimes were sufficient to eliminate all ALDH1A2 activity. An alternative protocol where on the day of birth and on 1 day of age the males were injected with tamoxifen produced more robust results. Under this protocol none of the males produced any sperm throughout their lifetime. Some of these mice treated with tamoxifen on day of birth and day 1 after birth were raised to 23 days of age and injected with a bolus of RA. The histology of the testes of these animals were examined 42 days after the injection of RA but the block at the conversion of A spermatogonia to A1 spermatogonia was still in place and no sperm were produced (**Figure 1**). This was expected since there is no source of RA from the germ cells or Sertoli cells to support spermatogenesis as was also seen for *Aldh1a1* ^-/-^, *Aldh1a2* ^fl/fl^, *Stra8-Cre+*, *Amh*-Cre+ (**Table 1** line 6).

**Table 1:** The Aldh1a1^-/-^, Aldh1a2^f/f^ mice were crossed with the designated Cre to delete gene in Sertoli cells or germ cells or both.

Experiment	N	Testis wt.	Sperm/cauda
control	10	0.129+/-.013	92+/-27
Stra8 *Cre*	7	0.118+/-.013	79.6+/-37
AMH *Cre*	6	0.018+/-.002	zero
AMH *Cre* +RA	9	0.077+/-0.018	84+/-21
Stra8 *CRE* and AMH *CRE*	8	0.025+/-.005	zero
Stra8 *CRE* and AMH *CRE* plus RA	7	0.029+/-.005	zero

In some experiments (4 and 6) mice were treated with RA and analyzed 4 weeks later to determine if spermatogenesis could recover. N is number of individual mice. Values plus standard deviation are shown.

The mice with globally deleted *Aldh1a1* and *Aldh1a2* from either tamoxifen injection protocol that resulted in aspermatogenesis were viable and appeared normal in all respects with the exception of the testis (**Table 2**). A detailed pathological examination was not done but the mice were routinely aged to 4 months and some were left for over 6 months and showed normal body weight and no obvious pathologies.

**Table 2:** The Aldh1a1^-/-^, Aldh1a2^f/f^ mice were crossed with the ERT Cre.

Experiment Aldh1a1^-/-^, Aldh1a2^f/f^	N	Testis wt. (mg)	Sperm/2 cauda (millions)
1. *ERT* CRE 8-10 postnatal	10	86+/-17	72.2+/-25.1
2. *ERT* CRE 21-25 postnatal	10	107+/-7	96+/-9.9
3. *ERT* CRE 8-10 postnatal and 21-25 postnatal	8	17+/-7	zero
4. *ERT* CRE 0-1 postnatal	7	11+/-3	zero
5. *ERT* CRE 0-1 d postnatal plus RA	7	29+/-5	zero

Tamoxifen injections were given on the designated days after birth to delete Aldh1a2 gene globally. In experiment 5 mice were treated with RA and analyzed 4 weeks later to determine if spermatogenesis could recover. N is number of individual mice. Values plus standard deviation are shown.

## Discussion

The action of retinoic acid (RA) is required for normal spermatogenesis in rodents and possibly all mammals (15). We have previously shown that RA is synthesized locally in pulses along the seminiferous tubules (16). These pulses are required for the transition of undifferentiated A spermatogonia into A1 spermatogonia and into the differentiation pathway (7). The location of these pulses corresponds to the onset of spermatogenesis and the initiation of the cycle of the seminiferous epithelium. In the absence of RA there is no cycle, and no germ cells advance beyond undifferentiated spermatogonia. It has been established that the pulse of retinoic acid is a result of the localized synthesis of retinal by retinol dehydrogenase 10 (RDH10) and the conversion of retinal to retinoic acid by 3 aldehyde dehydrogenases designated ALDH1A1, ALDH1A2 and ALDH1A3 (8, 9, 17, 18). Both the Sertoli cells and the germ cells have the capacity to synthesize RA (9).

Deletion of either the *Aldh1a1* gene or *Aldh1a2* gene alone has no major consequences to spermatogenesis or the mice. Teletin et al. (9) used a Cre-Lox P approach to eliminate all 3 *Aldh1a* genes from Sertoli cells or from germ cells or from both cell types. From these experiments they determined that RA from Sertoli cells was essential to begin the first wave of germ cell development. Deletion of all 3 genes from germ cells had no effect on spermatogenesis. However, in the Sertoli cell specific triple gene deletion, if RA was present during the first wave in the form of a single injection, spermatogenesis proceeded normally and was continuous suggesting that the germ cell RA was sufficient to maintain spermatogenesis once it had been initiated. We addressed these studies using a different genetic approach where we left the *Aldh1a3* gene intact. While there are only low levels of ALDH1A3 in the testis we wanted to determine if it was sufficient to maintain spermatogenesis.

Our cell specific deletions of only *Aldh1a1* and *Aldh1a2* recapitulated the results from Teletin et al. (9) who deleted all 3 *Aldh1a* genes. Deletion of these 2 genes and maintenance of the *Aldh1a3* gene in Sertoli cells completely blocked spermatogenesis unless an injection of RA was made. Thus, the presence of ALDH1A3 alone is not sufficient to maintain spermatogenesis. In addition, deletion of these two genes in germ cells had no effect on sperm production. RA synthesized in Sertoli cells is sufficient to initiate and maintain spermatogenesis while RA from germ cells can only maintain spermatogenesis after it has been initiated.

Because of the absolute requirement of RA for spermatogenesis, it has been proposed that inhibition of the synthesis or the action of RA could be a possible approach for contraceptive development (10, 11, 19). Blocking RA synthesis with an aldehyde dehydrogenase inhibitor or use of RA analogs that inhibit the action of retinoic acid receptors (RAR) have been shown to block spermatogenesis (10, 20–22). However, given the prevalence of the RA signaling system in biology and its absolute requirement in embryogenesis there was serious concern about developing a contraceptive approach for the testis that could have serious consequences for other organ systems. Previously it has been shown that global deletions of the genes for Cyp26A1 and Cyp26b1, the enzymes involved in RA homeostasis, lead to increased concentrations of RA in several organs, reduced lifespan, failure to gain weight, and fat atrophy (23). So, increased RA concentrations in adult mice led to severe physiological consequences. Therefore, one of the goals of this study was to examine the viability of mice after global deletion of 2 of the 3 *Aldh1a* genes and a decreased ability to synthesize RA. We found that the global deletion of *Aldh1a1* and *Aldh1a2* had no apparent effect on the gross viability of the mice. Teletin et al. (9), only reported data on the testis cell specific deletion of all 3 genes coding for ALDH1A enzymes so in our studies it is possible that ALDH1A3 was able to compensate in some tissues other than the testis. Nonetheless, inhibitors targeting ALDH1A1 and ALDH1a2 would certainly act as effective contraceptive compounds while not affecting gross viability. While we did not examine the physiopathology of potentially affected systems such as the immune system these results are significant in attesting to the feasibility of a RA focused contraceptive approach.

## Data Availability Statement

The original contributions presented in the study are included in the article/supplementary material. Further inquiries can be directed to the corresponding author.

## Ethics Statement

The animal study was reviewed and approved by WSU IACUC.

## Author Contributions

Experimental protocols were done by TT and the experiments were planned by TT and MG.

## Funding

Supported by HD 10808 from NIH.

## Conflict of Interest

The authors declare that the research was conducted in the absence of any commercial or financial relationships that could be construed as a potential conflict of interest.

The reviewer KR declared a shared affiliation with the authors to the handling editor at the time of review.

## Publisher’s Note

All claims expressed in this article are solely those of the authors and do not necessarily represent those of their affiliated organizations, or those of the publisher, the editors and the reviewers. Any product that may be evaluated in this article, or claim that may be made by its manufacturer, is not guaranteed or endorsed by the publisher.
